# The Immune Contexture in Canine Anal Sac Adenocarcinoma: Immunohistochemical Quantification of Tumor-Infiltrating Lymphocytes and Tumor-Associated Macrophages with Image Analysis

**DOI:** 10.3390/ani14243696

**Published:** 2024-12-20

**Authors:** Barbara Bacci, Barbara Brunetti, Cristiano Maino, Ginevra Martinoli, Nick J. Bacon, Giancarlo Avallone

**Affiliations:** 1 Department of Veterinary Medical Sciences, University of Bologna, via Tolara di Sopra 50, 40064 Bologna, Italy; barbara.bacci@unibo.it (B.B.); cristiano.maino@studio.unibo.it (C.M.); ginevra.martinoli2@unibo.it (G.M.); giancarlo.avallone@unibo.it (G.A.); 2 AURA Veterinary, 70 Priestley Road, Surrey Research Park, Guildford GU2 7AJ, UK; nickb@auravet.com; 3 Department of Veterinary Medicine, University of Surrey, Guildford GU2 7AL, UK

**Keywords:** anal sac, adenocarcinoma, CD3, CD20, Iba-1, FoxP3, lymphocytes, macrophages, tumor microenvironment, tumor-infiltrating lymphocytes (TILs), tumor-associated macrophages (TAMs)

## Abstract

Canine anal sac gland adenocarcinomas (ASACs) are locally aggressive and highly metastatic to regional lymph nodes. Tumor-infiltrating lymphocytes (TILs) and tumor-associated macrophages (TAMs) can be effective prognostic and predictive markers in numerous human neoplasms and are increasingly investigated in dogs. The aim of this study was to characterize immune cells in canine ASACs and their relationship with tumor size, histologic metastatic status, and tumor clinical stage. Thirty ASACs with known tumor size, metastatic status, and clinical stage were immunolabeled for Iba1 (macrophages), CD20 (B cells), CD3 (T cells), and Foxp3 (regulatory T cells). With image analysis, two areas of 1 mm^2^ were analyzed for each case at the tumor core (TC) and invasive margin (IM) and immune cells were counted. Eighteen patients had metastasis at the time of diagnosis, of which fifteen were nodal only, and three were both distant and nodal. The median tumor size was 32.5 mm (range 11–70). The clinical stage was I in five cases, II in seven cases, III in fifteen cases, and IV in three cases. T cells and macrophages were the most abundant immune cells in all tumors. Tumor size did not influence the number or type of infiltrating immune cells. By contrast, significantly higher numbers of TC T lymphocytes were found in patients without metastasis, while significantly higher numbers of TC macrophages were found in dogs with metastasis. Immune cell infiltrate did not differ according to clinical stage. The results indicate that the tumor immune microenvironment, specifically TILs and TAMs, contribute to tumor behavior and may influence metastatic potential; in particular, high CD3 infiltration may prevent tumor progression, while increased macrophage infiltration could promote it.

## Simple Summary

Anal sac gland adenocarcinoma is an aggressive neoplasm in dogs; surgical excision is the main treatment, but its success is often compromised by the early tendency of the tumor to metastasize. The immune contexture, that is, the density and distribution of immune cells within the tumor, has significant prognostic value in several human neoplasms. In this study, we investigated whether immune cells may affect the clinical behavior of ASACs. To achieve this, we analyzed the quantity and distribution of B, T, and T regulatory lymphocytes, macrophages in a series of ASACs, to verify potential differences based on primary tumor size, metastatic status, and clinical stage at the time of diagnosis. Higher numbers of T cells and lower numbers of macrophages were found in tumors with metastasis, while no differences were found according to tumor size and clinical stage. This could indicate a role of T cells and macrophages in tumor progression and could be further investigated for immunotherapy purposes.

## 1. Introduction

Anal sac gland adenocarcinoma (ASAC) in dogs is a highly aggressive cancer, in which metastatic disease is reported frequently at the time of diagnosis. Metastases typically begin in the sublumbar lymph nodes and can later spread to the spleen, liver, lungs, and other organs. The presence of metastasis at the time of initial diagnosis is a negative prognostic factor for ASACs. This was confirmed by several studies in the literature [1,2,3]. Tumor size is also a critical prognostic factor and several cutoff values are proposed in the literature, ranging from 2 to 10 cm [3,4,5]. Polton et al. initially proposed a cutoff of 2.5 cm within the proposal of a clinical staging system [1], and its prognostic value was later confirmed by other authors [6]. Specifically, tumor size > 2.5 cm was significantly associated with metastasis at presentation in one study [6]. More recently, a cutoff of 2 cm was proposed, with findings indicating that 20% of dogs with tumors smaller than 2 cm had metastasis, compared to 63% in those with larger tumor sizes [7].

However, other studies do not report significant associations between tumor size and survival [3,6]. Clinical stage, determined by combinations of tumor size and metastasis, is also significantly associated with outcome according to some authors [1] but not others [4,8]. In addition to clinical characteristics, various histological and immunohistochemical parameters have been studied as potential prognostic indicators, with poorer outcomes linked to tumors exhibiting solid growth patterns, necrosis, and lymphovascular invasion [2,3,4]. Although mitotic count has been investigated as a potential prognostic factor in a number of studies, this parameter could not consistently predict outcomes [2,3,4].

Over the past two decades, researchers have explored the intricate relationships between tumors and the immune system in human cancers, leading to significant progress in prognostic evaluations and treatments. It is known that tumor-infiltrating immune cells can influence tumor progression and metastasis. While the neoplastic microenvironment can favor chronic pro-tumorigenic inflammatory states, neoplastic cells also have the ability to escape immune-surveillance mechanisms [9]. Tumor-infiltrating lymphocytes (TILs) have an important role in promoting anti-tumor immunity in a variety of solid tumors in humans, in particular in breast, ovarian, and non-small-cell lung cancer [10]. Tumor-associated macrophages (TAMs) are another fundamental component of tumor-infiltrating immune cells and exhibit two polarization states in response to different microenvironmental signals—M1 and M2. M1 macrophages are pro-inflammatory, while M2 macrophages have an immunosuppressive phenotype; hence, M2 infiltrates are typically associated with unfavorable outcomes [11].

Based on the degree of lymphocytic infiltration and molecular signatures, tumors can be classified into three different phenotypes: inflamed, immune-excluded, and immune-deserted. Immune-inflamed tumors, also known as “hot” tumors, are characterized by high levels of T-cell infiltration and are more likely to benefit from immunotherapy. Conversely, tumors transitioning towards immune-excluded and immune-deserted states are termed as “cold” tumors. Immune-excluded tumors confine CD8^+^ T lymphocytes to the periphery, preventing their infiltration into the central tumor mass. On the other hand, immune-deserted tumors lack CD8^+^ T lymphocytes both within the tumor and in its surroundings [12,13,14].

Immune cells are represented by TILs, a diverse group that includes CD8^+^ T lymphocytes, which have direct cytotoxic activity, and FoxP3+ regulatory T cells (Tregs), which can suppress immune responses. B cells seem to have a prognostically favorable effect, although their role was associated with negative prognosis in other studies. Finally, TAMs have been linked to poorer prognosis due to their role in promoting angiogenesis and secreting cytokines that promote invasion [10].

Limited information is available on the immune cell infiltrate in ASACs and mainly consists of one study in which CD3+ and CD20+ infiltrates as well as PD-1/PD-L1 expression were investigated. Results indicated a dominant CD3+ infiltrate, supporting an inflamed phenotype, and a variable PD-1/PDL1 expression which influenced survival in dogs treated by surgery alone [15].

To expand the knowledge on the immune contexture in ASACs, the extent and distribution of TILs and TAMs, CD3+ lymphocytes, CD20+ B lymphocytes, FoxP3+ T regulatory lymphocytes, and Iba-1+ macrophages were analyzed at the invasive margin and in the center of the tumor, and the results were correlated with clinical variables of tumor size, metastatic status, and clinical stage at the time of diagnosis.

## 2. Materials and Methods

### 2.1. Case Selection

Thirty client-owned dogs treated for ASAC at Fitzpatrick Referrals Oncology and Soft Tissue, Surrey, now AURA Veterinary (UK), between 2016 and 2019, were included. Dogs that underwent a surgical excision of the primary tumor and had a histopathological diagnosis of ASAC of both primary tumors and metastasis were included in the study. Histological diagnosis was confirmed in all cases. Formalin-fixed paraffin-embedded (FFPE) cases were retrieved and multiple sections were cut from each block of the primary tumor for immunohistochemical analysis. Histological, immunohistochemical, and digital image analysis (DIA) were conducted at the University of Bologna, Department of Veterinary Medical Sciences (DIMEVET). Tumor size values were retrieved from sample trimming data and the maximum diameter was reported. Histologically confirmed lymph node and distant organ metastasis were evaluated as present or absent, and the site of metastasis was recorded for each case. Clinical stage was also available for each patient, according to the scheme proposed by Polton et al., 2007 [1].

### 2.2. Immunohistochemical Methods

All cases underwent immunohistochemical (IHC) assessment with antibodies for CD3, CD20, FoxP3, and Iba-1. Three-micrometer-thick sections were dewaxed and rehydrated. Endogenous peroxidase was blocked by immersion in 3% H_2_O_2_ in methanol for 30 min. Source, dilution, and retrieval protocols for each antibody are reported in Table 1. The reaction was amplified by the avidin–biotin method (Vectastain^®^ Elite ABC-HRP kit, Vector, Burlingame, CA, USA) and visualized with 0.04% 3,3′-diaminobenzidine (Code: 10-0048, Histoline, Milano, Italy) for 4 min. Sections were counterstained with hematoxylin, rinsed in tap water, and dehydrated, before a coverslip was added. Sections of canine hyperplastic lymph node were used as positive controls. Negative controls comprised slides incubated with omission of the primary antibody and internal negative controls constituted by the neoplastic cells of the same samples known to be non-reactive for the specific antibody. The immunohistochemical slides were scanned with Grundium Ocus^®^ 20 (Tampere, Finland) at 20× magnification (0.25 μm/pixel) to obtain a whole-slide image (WSI). The digital images were analyzed with the open-source DIA software QuPath v0.5.0.3 [16].

### 2.3. Digital Image Analysis

**Figure 1:**
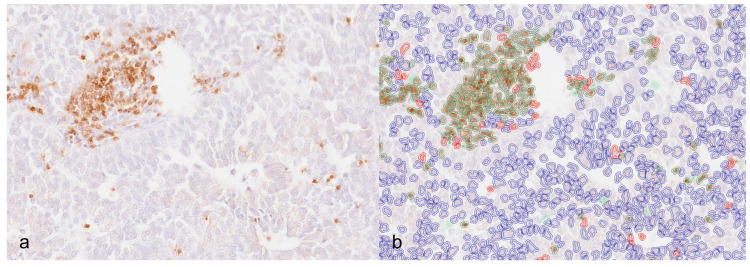
Canine anal sac adenocarcinoma. (**a**) Immunohistochemistry for CD3 where scattered lymphocytes are visible both in aggregates and isolated. (**b**) Image analysis in the same area showing CD3-positive lymphocytes circled in red, tumor/negative nuclei circled in blue, and stromal nuclei (positive and negative) in green.

For each marker, positive cells were counted in WSI using DIA. For each case, 1 mm^2^ was manually searched and drawn in the tumor core (TC) and at the invasive margin (IM). The tumor core area was chosen from the inner tumor mass, while the IM area was chosen from the periphery and included both the tumor and the peripheral tissue. For the selection of both areas, slides were scanned at low power and the regions with a higher number of positive cells were chosen, avoiding areas of necrosis, hemorrhage, and artefacts. Automatic cell counting was performed using the positive cell detection tool. As the intensity of labeling varied within the cohort, the immunohistochemical and hematoxylin stain estimates for each digitized slide were adjusted (estimate stain vector tool). Analysis was performed, starting with manual selection of the whole section area and the use of the positive cell detection tool (Analyze → Cell analysis → Positive cell detection → adjust parameters based on Optical Density Sum; Score compartment: Nucleus DAB OD Mean). Default parameters for cell detection were adjusted to best match the outline of lymphocytes/macrophages and the results for each case were visually checked by the operator for satisfactory quality. To separate positive nuclei of tumors and stroma, the object classifier tool was applied. A random forest object classifier was created by identifying representative tumor cells and stromal cells and applied to the whole section to separate stromal and tumoral detected nuclei (Figure 1a,b).

CD3+, CD20+, FoxP3+, and Iba-1cell densities were calculated as the number of cells/mm^2^, as previously reported [17]. The B-cell/T-cell ratio and the percentage of T cells being FoxP3+ were also calculated. Each parameter was also reported for the intraepithelial (IE) compartment and stroma (S) separately.

### 2.4. Statistical Analysis

Statistical analysis was performed with R version 4.2.0. Mean and Standard Deviation (SD) were calculated for normally distributed data, while median (min–max) was reported for non-normally distributed data. Associations were determined with Chi-square tests for categorical variables and Kruskal–Wallis tests or one-way ANOVA for continuous variables. Values < 0.05 were considered statistically significant.

## 3. Results

### 3.1. Patients Data

In total, 14/30 dogs were females (of which 1 was spayed) and 16/30 were males (of which 4 were neutered). The mean age was 6.66 ± 3.2, in a range of 1–11 years. The most represented breeds were Labrador Retrievers (10/30) and Cocker Spaniels (4/30).

Eighteen patients had metastasis at the time of diagnosis, of which fifteen were nodal only, and three were both distant and nodal.

Tumor size ranged from 11 to 70 mm, with a median size of 32.5 mm. When applying the cutoff value used for clinical stage (25 mm), 10 cases were ≥25 mm and 20 cases were below the cutoff. Clinical stage was also recorded and was I in five cases, II in seven cases, III in fifteen cases (of which ten were IIIa and five were IIIb), and IV in three cases.

### 3.2. Immunohistochemistry and Digital Image Analysis

**Figure 2:**
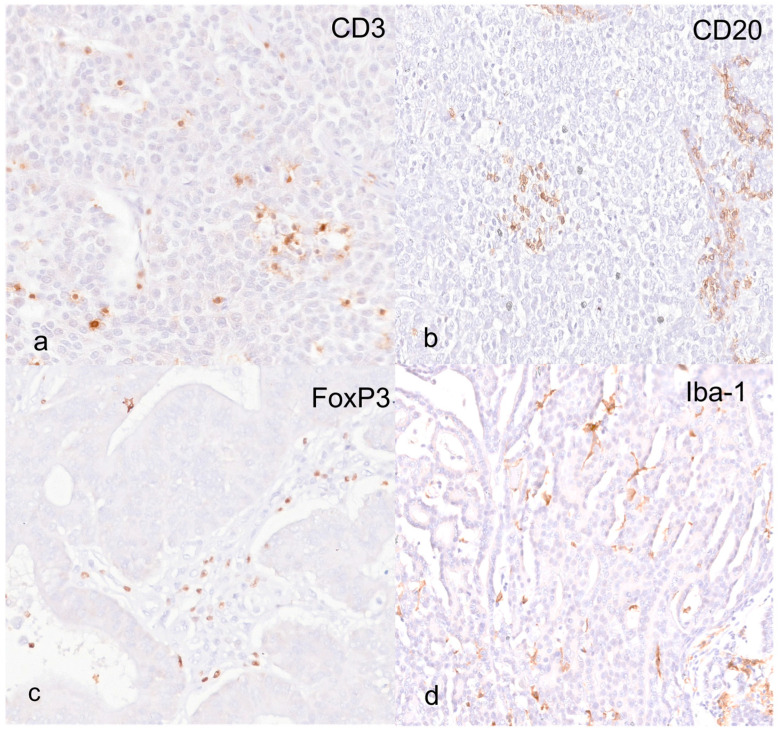
Canine anal sac adenocarcinoma. (**a**) Immunohistochemistry for CD3; scattered CD3-positive lymphocytes within the tumor, both intraepithelial and in the fine fibrovascular stroma. (**b**) Immunohistochemistry for CD20. Scattered CD20-positive lymphocytes within the tumor, both intraepithelial and in the fine fibrovascular stroma. (**c**) Immunohistochemistry for FoxP3; scattered T-regulatory lymphocytes confirmed within the tumor stroma. (**d**) Immunohistochemistry for Iba-1; scattered macrophages are visible in both the intraepithelial and stromal compartments.

The total CD3+ T cells detected at the tumor core (TC) and invasive margin (IM) were similar; specifically, the median values were 521 cells/mm^2^ (57–2696 cells/mm^2^) and 534 cells/mm^2^ (38–3238 cells/mm^2^), of which the vast majority were localized in the tumor stroma (Table 2; Figure 2a).

Total FoxP3+ Tregs were higher in the IM than in the TC (172 cells/mm^2^ [25–453 cells/mm^2^] versus 93 cells/mm^2^ [17–705 cells/mm^2^]), and in both areas, the majority of Tregs were found within the stromal compartment, while very few cells were detected in the intraepithelial (IE) compartment (Table 2; Figure 2c).

CD20+ B cells were detected in slightly higher numbers in the IM than in the TC, with the majority of B lymphocytes being localized in the stroma (Table 2; Figure 2b); however, this difference was not statistically significant.

Iba-1+ macrophages were the most abundant infiltrate in both TC and IM areas, where they were detected in similar numbers. Of the total macrophage median count, which was 1089 cells/mm^2^ (156–3569 cells/mm^2^) in the TC and 1004 cells/mm^2^ (173–3350 cells/mm^2^) in the IM, the higher percentage was counted in the stromal compartment (Table 2; Figure 2d).

The FoxP3/CD3 ratio was also calculated to assess which proportion of CD3+ cells were FoxP3+. Within the CT, the median ratio was 0.19 (0.01–391), while at the IM, the ratio was 0.27 (0.04–4.00).

### 3.3. Clinical/Immunohistochemical Associations

**Figure 3:**
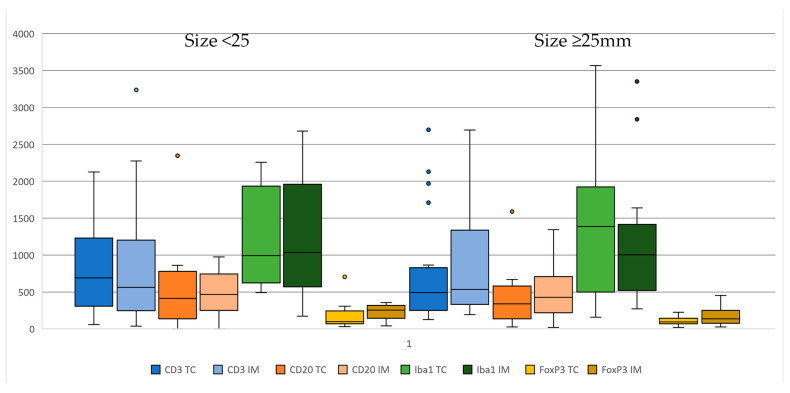
Boxplots showing immune cell infiltrates according to size, with cutoff set at 25 mm (**left** < 25 mm, **right** ≥ 25 mm). Immune cells are CD3+ T cells, CD20+ B cells, Iba-1+ macrophages, and FoxP3+ T cells (**left** tumor core (CT) and **right** invasive margin (IM)).

TIL and TAM numbers were analyzed in relation to the presence of metastasis (nodal only and nodal + distant), tumor size, and clinical stage. Immune cell density did not vary significantly according to tumor size. Although all lymphocytes tended to be higher in tumors below 25 mm and higher in those above the cutoff, differences were not statistically significant (Figure 3).

**Figure 4:**
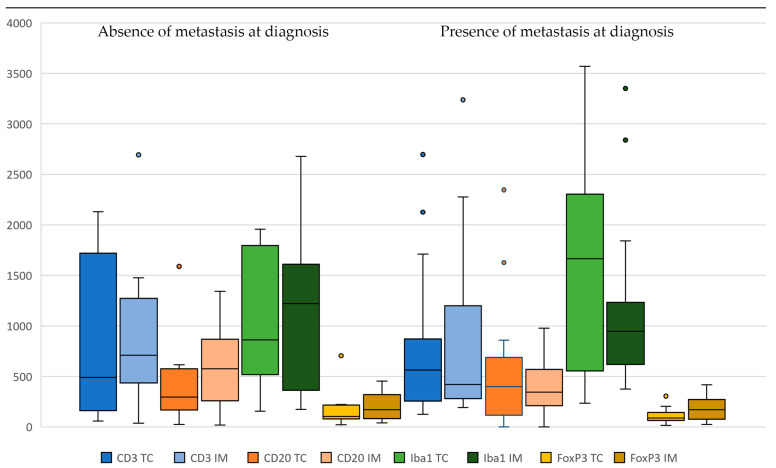
Boxplots showing immune cell infiltrates according to absence (**left**)/presence (**right**) of metastasis at diagnosis. Immune cells are CD3+ T cells, CD20+ B cells, Iba-1+ macrophages, and FoxP3+ T cells (**left** tumor core (CT) and **right** invasive margin (IM)).

Immune cell quantification was also assessed based on the presence of metastasis at diagnosis. All CD3+ lymphocytes were in similar amounts in cases with and without metastasis. However, when the IE compartment and stroma were analyzed separately, IE T cells were significantly different in dogs without metastasis compared to those with metastasis, both in the TC (121.50 cells/mm^2^ [19.00, 1882.00 cells/mm^2^] versus 10.00 cells/mm^2^ [0.00, 1310.00 cells/mm^2^]) and in the IM (174.00 cells/mm^2^ [6.00, 2030.00 cells/mm^2^] versus 9.00 cells/mm^2^ [0.00, 2949.00 cells/mm^2^]) (*p* = 0.016 and 0.008, respectively) (Figure 4). Separate analysis of the IE/stromal compartments was carried out for all other TILs, but no significant differences were found. No significant differences were found in total TAMs based on metastatic status. However, when stromal and IE compartments were analyzed separately, both in the CT and at the IM, tumor macrophages were markedly higher in the patients with metastasis compared to the ones without metastasis (8.00 cells/mm^2^ [0.00–267.00 cells/mm^2^] versus 198.50 cells/mm^2^ [0.00–2956.00 cells/mm^2^] in TC and 8.50 cells/mm^2^ [0.00–293.00 cells/mm^2^] versus 50.00 [0.00–1229.00 cells/mm^2^] in the IM) (*p* = 0.070 and 0.048, respectively) (Table 3).

**Figure 5:**
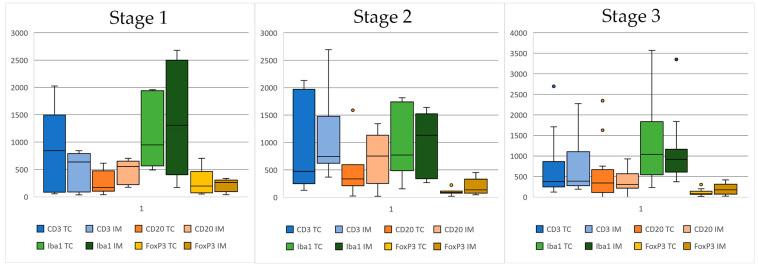
Boxplots showing immune cell infiltrates according to clinical stage. Due to the low number of stage 4 cases, these are not plotted in this figure. Immune cells are CD3+ T cells, CD20+ B cells, Iba-1+ macrophages, and FoxP3+ T cells (**left** tumor core (CT) and **right** invasive margin (IM)).

Regarding clinical stage, the immune infiltrate did not differ significantly across stage groups, although when stroma and IE were evaluated separately, CD3+ T cells were markedly different according to clinical stage, for both TC and IM. In fact, T cells were markedly lower in stages 3 and 4 compared to stages 1 and 2, with median counts being as follows for TC: stage I: 52.00 cells/mm^2^ [19.00–329.00 cells/mm^2^]; stage II: 139.00 cells/mm^2^ [19.00–1882.00 cells/mm^2^]; stage III: 12.00 cells/mm^2^ [0.00–685.00 cells/mm^2^]; stage IV: 2.00 cells/mm^2^ [0.00–1310.00 cells/mm^2^] (*p* = 0.087). For IM, the median counts were as follows: stage I: 86.00 cells/mm^2^ [8.00–559.00 cells/mm^2^]; stage II: 498.00 [6.00–2030.00]; stage III: 10.00 [0.00–472.00]; stage IV: 1.00 [0.00–2949.00] (*p* = 0.048) (Figure 5).

The results are summarized in Supplementary Table S1.

## 4. Discussion

This study evaluates the presence, amount, and distribution of immune cells in canine anal sac adenocarcinoma in order to understand the role of TILs and TAMs in relation to clinical disease at the time of diagnosis. TILs and TAMs have been investigated in a number of canine tumors, specifically in melanocytic neoplasms [18,19,20,21], mast cell tumors [22], mammary tumors [23,24,25], and soft tissue sarcomas [26], amongst others.

In the present study, T lymphocytes and macrophages were the most abundant populations, while B cells were the least numerous. All immune cells were more abundant in the stromal compartment than in the intraepithelial areas, both at the tumor core and in the invasive margin, suggesting that ASACs can be considered inflamed (hot) immune types, as also found in previous studies [15].

In our study on ASACs, macrophages were the most abundant immune cell infiltrate. TAMs were investigated in other canine tumors with different results. In canine mammary carcinomas, high levels of TAMs were related to poor prognosis [25]. Similarly, high numbers of TAMs were observed in dogs with high-grade sarcomas, compared to low-grade tumors, confirming their association with negative prognostic factors [26].

T cells were also found in high numbers in the present study. High T-cell infiltrate levels were also found in canine mast cell tumors, where they have been linked to the prevention of nodal metastasis development [22]. T-cell infiltration was also found to be prognostically relevant in canine mammary carcinomas; however, T-cell populations were assessed by flow cytometry [24]. In our study, B cells were the least numerous, which is in accordance with other works in canine mammary carcinomas and mast cell tumors [22]. However, B cells were found to be the most abundant lymphocytes in canine melanomas, and their infiltration was associated with tumor-related death, presence of metastasis/recurrence, shorter overall and disease-free survival, and increased hazard of death and development of recurrence/metastasis [18].

Tumor-infiltrating lymphocytes can be evaluated depending on their location as lymphocytes within cancer cell nests (intraepithelial lymphocytes), lymphocytes in the central cancer stroma (stromal lymphocytes), and lymphocytes present along the invasive margins. Conflicting results are available in the literature on the roles of IE and stromal TILs; some authors claim that stromal lymphocytes have greater prognostic implications due to their ability to promote anti-tumor activity by cytokine secretion [9], while others indicate that the proximity of lymphocytes to tumor cells may favor their direct cytotoxic effect on tumor cells, hence having greater significance [27].

Immune cell counts were analyzed here in relation to clinical variables of tumor size, presence of metastasis at the time of diagnosis, and clinical stage. Negligible differences in T-cell and B-cell numbers were observed according to both tumor size and metastatic status. However, when IE and stromal compartments were analyzed separately, high T lymphocyte infiltrates within the IE compartment demonstrated a significant association with metastatic status; in fact, patients with metastasis at the time of diagnosis had significantly lower IE T-cell infiltrates. To explain this finding, there is increasing evidence that T cells, more specifically CD8^+^ lymphocytes, have anti-tumoral activity due to their cytotoxic effect. In fact, resistance to cancer immunotherapy has been linked to defective T-cell migration from the stroma into the tumor [27].

In addition to higher lymphocytes, dogs with metastasis at the time of diagnosis also had a markedly higher number of macrophages in both the intraepithelial and stromal compartments, suggesting that macrophages could have a pro-metastatic effect. Previous studies in the literature found that poor outcome was associated with high macrophage infiltrates in numerous human and canine neoplasms, probably due to their role in producing cytokines, chemokines, and growth factors that facilitate tumor growth and invasion [22,28,29,30]. Overall, the concurrent lower lymphocytic infiltrate and higher macrophage content within the intraepithelial compartment in patients with metastasis may be explained by a mechanism of immune evasion that interferes with the migration of T cells from the stroma to the tumor itself and concurrent macrophage influx that promotes tumor progression [9,27].

Amongst the immune cells that were investigated, Tregs are known for their immune-suppressive effect and are therefore more often associated with poor outcomes in different human tumors [31]. The link between increased intratumoral Tregs and poor prognosis has been established in other canine cancers, including oral malignant melanoma [32]. However, in the present study, Tregs and the Treg/CD3 ratio were not found to be associated with any of the clinical variables investigated; therefore, their roles and clinical significance need to be further investigated in ASACs.

Since the evaluation of TILs is not standardized for veterinary patients, identification of consistent methods of analysis for each tumor type are needed to allow comparisons across published data and to establish more reliable methods to link prognosis with tumor microenvironment. Specifically, guidelines for area selection within the tumor and standardization of scoring systems for immune cell infiltrates would be needed for each tumor type in order to provide clinicians with additional prognostic information and therapeutic options. In addition, our study suggests that for future studies on the prognostic role of immune cells in ASACs, separate analysis of tumor and stroma may prove to be more informative than total cell counts.

Digital image analysis is an increasingly used method to evaluate immunohistochemical positivity, which has the advantage of producing more reliable and reproducible data. The ability of image analysis software to quantify immune cells accurately and to perform separate counts for stroma and tumors separately may be explored in the future to establish standardized scoring systems for the immune microenvironment.

## 5. Conclusions

This study confirms that ASACs are characterized by a “hot” immune environment and that intratumoral TILs are more numerous in patients with small tumor size and absence of metastasis, indicating a potentially beneficial role in preventing tumor progression. In parallel, increased intratumoral macrophages are found in dogs with larger tumor size and metastasis, suggesting a possible role of macrophages in promoting tumor growth and metastasis. Future studies will be aimed at evaluating the prognostic impact of the immune infiltrate in dogs with ASACs.

## Figures and Tables

**Table 1:** Details of the antibodies and protocols used for immunohistochemistry.

Antibody	Manufacturer/Code	Antigen Retrieval	Dilution
CD3	Dako, Glostrup, Denmark)/F7.2.38	EDTA pH 8.00	1:200
CD20	Invitrogen, Rockford, IL, USA/PAS16701	Citrate pH 6.00	1:1000
FoxP3	eBioscience, Invitrogen, Carlsbad, CA, USA/FJK-16S	Citrate pH 6.00	1:1000
Iba-1	Novus Biological, Centennial, CO, USA/NB100-1028	Citrate pH 6.00	1:200

**Table 2:** Median counts (cells/mm^2^) and range of CD3+ T cells, FoxP3+ Tregs, CD20+ B cells, and Iba-1+ macrophages, in the tumor core (CT) and invasive margin (IM), intraepithelial (IE) compartment, and stroma (S).

Tumor Core (Cells/mm^2^) Median (Range)				Invasive Margin (Cells/mm^2^) Median (Range)		
	Total	IE	S	Total	IE	S
CD3+	521 (57–2696)	47.5 (0–1882)	353.5 (2–2693)	534 (38–3238)	51.5 (5–25–2949)	347 (9–2176)
FoxP3+	93 (17–705)	7.50 (0–127)	75 (12–627)	172 (25–453)	19 (0–188)	138 (24–374)
CD20+	339 (0–2346)	1 (0–269)	342 (23–2346)	428 (0–1342)	3 (0–387)	467 (19–1117)
Iba-1	1089 (156–3569)	92 (0–2956)	881 (150–2751)	1004 (173–3350)	24 (0–1229)	781 (173–2588)

**Table 3:** Median counts (cells/mm^2^) and range of intraepithelial (IE) CD3+ T cells and Iba-1+ macrophages in the tumor core (CT) and invasive margin (IM), in patients with metastasis at diagnosis.

Immune Cell		Metastasis at Diagnosis Cells/mm^2^ [Range]		
		No	Yes	*p*-Value
IE-CD3+	CT	121.50 [19.00–1882.00]	10.00 [0.00–1310.00]	0.016
	IM	174.00 [6.00–2030.00]	9.00 [0.00–2949.00]	0.008
IE-Iba-1	CT	8.00 [0.00–267.00]	198.50 [0.00–2956.00]	0.070
	IM	8.50 [0.00–293.00]	50.00 [0.00–1229.00]	0.048

## Data Availability

The original contributions presented in the study are included in the article; further inquiries can be directed to the corresponding author.

## Supplementary Materials

The following supporting information can be downloaded at https://www.mdpi.com/article/10.3390/ani14243696/s1, Table S1: Immune cell quantification in 30 ASACs (CD3+ T-cells, CD20+ B-cells, FoxP3+ T cells, and Iba-1+ macrophages). Cells are quantified in two separate areas (TC—tumor core and IM—invasive margin), each area evaluated in 1 mm^2^.
