# Supplementary material for: The Immune Contexture in Canine Anal Sac Adenocarcinoma: Immunohistochemical Quantification of Tumor-Infiltrating Lymphocytes and Tumor-Associated Macrophages with Image Analysis
Source: Animals (Basel). 2024 Dec 20;14(24):3696. doi: 10.3390/ani14243696 (PMC11672758; doi:10.3390/ani14243696)
Supplement: Supplementary file 1 [file animals-14-03696-s001.zip › Table S1.pdf]

Supplementary Table S1

Immune cell quantification in 30 ASACs (CD3+ T-cells, CD20+ B-cells, FoxP3+ T cells, and Iba-1+ macrophages). Cells are quantified in two separate areas (TC- tumor core and IM-invasive margin), each area evaluated in 1 mm^2

cells/mm^2 [range]	Size ≥25mm		Metastasis at diagnosis		Clinical stage			
**Immune cell**	no	yes	no	yes	I	II	III	IV
**CD3+ TC**	689.00 [57.00-2126.00]	491.50 [126.00-2696.00]	491.50 [57.00-2130.00]	563.00 [126.00-2696.00]	843.00 [57.00-2025.00]	476.00 [132.00-2130.00]	381.00 [126.00-2696.00]	723.00 [595.00-2126.00]
**CD3+ IM**	560.50 [38.00-3238.00]	534.00 [192.00-2694.00]	709.50 [38.00-2694.00]	420.00 [192.00-3238.00]	638.00 [38.00-844.00]	747.00 [372.00-2694.00]	387.00 [192.00-2276.00]	542.00 [326.00-3238.00]
**FoxP3+ TC**	96.00 [28.00-705.00]	93.00 [17.00-224.00]	104.50 [23.00-705.00]	87.50 [17.00-306.00]	198.00 [52.00-705.00]	93.00 [23.00-224.00]	83.00 [17.00-306.00]	115.00 [28.00-145.00]
**FoxP3+ IM**	251.50 [39.00-355.00]	135.50 [25.00-453.00]	172.00 [39.00-453.00]	172.50 [25.00-419.00]	262.00 [39.00-337.00	138.00 [49.00-453.00]	181.00 [25.00-419.00]	164.00 [76.00-224.00]
**CD20+ TC**	413.50 [0.00-2346.00]	339.50 [24.00-1627.00]	296.00 [24.00-1588.00]	296.00 [24.00-1588.00]	172.00 [39.00-614.00]	337.00 [24.00-1588]	344.00 [0.00-2346.00]	654.00 [105.00-859.00]
**CD20+ IM**	465.50 [0.00-976.00]	428.50 [20.00-1342.00]	576.00 [20.00-1342.00]	343.50 [0.00-976.00]	554.00 [174.00-706.00]	752.00 [20.00-1342.00]	310.00 [0.00-930.00]	470.00 [188.00-976.00]
**Iba-1+ TC**	993.50 [490.00-2258.00]	1387.00 [156.00-3569.00]	860.50 [156.00-1958.00]	1666.50 [236.00-3569.00]	948.00 [490.00-1958.00]	773.00 [156.00-1815.00]	1039.00 [236.00-3569.00]	2441.00 [2258.00-2611.00]
**Iba-1+ IM**	1034.50 [173.00-2678.00]	1004.00 [270.00-3350.00]	1220.50 [173.00-2678.00]	947.00 [376.00-3350.00]	1307.00 [173.00-2678.00]	1134.00 [270.00-1638.00]	921.00 [376.00-3350.00]	973.00 [804.00-2839.00
